# Impact of oral care with versus without toothbrushing on the prevention of ventilator-associated pneumonia: a systematic review and meta-analysis of randomized controlled trials

**DOI:** 10.1186/cc11675

**Published:** 2012-10-12

**Authors:** Wan-Jie Gu, Yi-Zhen Gong, Lei Pan, Yu-Xia Ni, Jing-Chen Liu

**Affiliations:** 1 Department of Anaesthesiology, the First Affiliated Hospital of Guangxi Medical University, 22 Shuangyong Road, Nanning 530021, Guangxi, China; 2 Department of Evidence-based Medicine, the First Affiliated Hospital of Guangxi Medical University, 22 Shuangyong Road, Nanning 530021, Guangxi, China; 3 State Key Laboratory of Respiratory Disease, Guangzhou Medical College, 151 Yanjiang Road, Guangzhou 510120, Guangdong, China

## Abstract

**Introduction:**

Ventilator-associated pneumonia (VAP) remains a common hazardous complication in mechanically ventilated patients and is associated with increased morbidity and mortality. We undertook a systematic review and meta-analysis of randomized controlled trials to assess the effect of toothbrushing as a component of oral care on the prevention of VAP in adult critically ill patients.

**Methods:**

A systematic literature search of PubMed and Embase (up to April 2012) was conducted. Eligible studies were randomized controlled trials of mechanically ventilated adult patients receiving oral care with toothbrushing. Relative risks (RRs), weighted mean differences (WMDs), and 95% confidence intervals (CIs) were calculated and heterogeneity was assessed with the I^2 ^test.

**Results:**

Four studies with a total of 828 patients met the inclusion criteria. Toothbrushing did not significantly reduce the incidence of VAP (RR, 0.77; 95% CI, 0.50 to 1.21) and intensive care unit mortality (RR, 0.88; 95% CI, 0.70 to 1.10). Toothbrushing was not associated with a statistically significant reduction in duration of mechanical ventilation (WMD, -0.88 days; 95% CI, -2.58 to 0.82), length of intensive care unit stay (WMD, -1.48 days; 95% CI, -3.40 to 0.45), antibiotic-free day (WMD, -0.52 days; 95% CI, -2.82 to 1.79), or mechanical ventilation-free day (WMD, -0.43 days; 95% CI, -1.23 to 0.36).

**Conclusions:**

Oral care with toothbrushing versus without toothbrushing does not significantly reduce the incidence of VAP and alter other important clinical outcomes in mechanically ventilated patients. However, the results should be interpreted cautiously since relevant evidence is still limited, although accumulating. Further large-scale, well-designed randomized controlled trials are urgently needed.

## Introduction

Ventilator-associated pneumonia (VAP) is defined as pneumonia that occurs in patients receiving mechanical ventilation and that arises more than 48 to 72 hours after endotracheal intubation [1]. VAP remains one of the most common nosocomial infections in the intensive care unit (ICU) and affects 8% to 28% of patients receiving mechanical ventilation [2]. Although many studies have tried to assess the mortality attributable to VAP, it remains unclear. Moreover, VAP has been associated with prolonged duration of mechanical ventilation, longer ICU stays, and higher health-care costs [3-5]. Given the clinical consequences attributable to VAP, prevention of VAP is of great importance and is a priority in ICU care.

The main important mechanism for the development of VAP is aspiration of colonized oropharyngeal secretions into the lower respiratory tract [6]. The oral cavity is a potential reservoir for bacteria and provides a habitat for microorganisms responsible for VAP, so strategies to reduce bacteria in the oral cavity may decrease the development of VAP and improve oral hygiene [7]. Oral care with chlorhexidine solution has been found to reduce the risk of VAP, according to some published meta-analyses [8-10]; however, the role of oral care with toothbrushing has received scant attention and remains unclear. Nowadays, there are published randomized controlled trials (RCTs) regarding the effect of oral care with toothbrushing on the prevention of VAP. However, these studies have a modest sample size and convey inconclusive results. So we undertook a systematic review and meta-analysis of RCTs to assess the effects of oral care with toothbrushing on the incidence of VAP and other important clinical outcomes in adult critically ill patients receiving mechanical ventilation.

## Materials and methods

### Literature search and inclusion criteria

Relevant RCTs were identified by searching PubMed and Embase databases. Other websites, including Cochrane Central Register of Controlled Trials, Google Scholar, Chinese Biomedical Literature on disc, and http://ClinicalTrials.gov (up to July 2012), were also searched. The structured search strategies used the following format of search terms: ('toothbrushing' or 'tooth brushing' or 'dental' or 'teeth brushing' or 'brushing tooth' or 'brushing teeth') *and *'pneumonia'. The search was limited to human subjects and RCTs. No language restriction was imposed. We also manually checked the reference lists of RCTs to include other potentially eligible trials. This process was performed iteratively until no additional articles could be identified. The following inclusive selection criteria were applied: (a) study design: RCT; (b) study population: adult critically ill patients receiving mechanical ventilation; (c) intervention: oral care with toothbrushing (regardless of approach and liquid applied); (d) comparison intervention: oral care without toothbrushing; and (e) outcome measure: the incidence of VAP.

### Data extraction and outcome measures

Two authors (W-JG and LP) independently extracted the following data from each RCT: first author, publication year, number of patients (intervention/control), type of ICU/study population, severity of illness at ICU admission (intervention/control), study design, intervention group (oral care with toothbrushing), control group (oral care without toothbrushing), definition of VAP, the incidence of VAP, and other important clinical outcome data. Extracted data were entered into a standardized Excel file (Microsoft Corporation, Redmond, WA, USA) and were checked by a third author (Y-ZG). When the same population was reported in several publications, we retained only the most informative article or complete study to avoid duplication of information. Any disagreements were resolved by discussion and consensus. The primary outcome was the incidence of VAP. Secondary outcomes included ICU mortality, duration of mechanical ventilation, length of ICU stay, antibiotic-free days, and mechanical ventilation-free day.

### Quality scoring and risk-of-bias assessment

The methodological quality of each trial was evaluated by using the Jadad scale [11]. This tool places emphasis on the following three areas when defining the quality of an RCT: (a) randomization, (b) double-blinding, and (c) description of withdrawals and drop-outs. A score of 1 is given for each of the areas described. A further point is obtained where the method of randomization or blinding (or both) is given and is appropriate; where it is inappropriate, a point is deducted. The studies are said to be of low quality if the Jadad score is not more than 2 and of high quality if the score is at least 3 [12].

Risk-of-bias assessment was performed in accordance with guidelines outlined in the *Cochrane Handbook for Systematic Reviews of Interventions *(version 5.1.0) [13]. Two authors subjectively reviewed all studies and assigned a value of ' high', 'low', or 'unclear' to the following: (a) selection bias (Was there adequate generation of the randomization sequence? Was allocation concealment satisfactory?); (b) performance and detection bias (Was there blinding of participants, personnel, and outcome assessors?); (c) attrition bias (Were incomplete outcome data sufficiently assessed and dealt with?); (d) reporting bias (Was there evidence of selective outcome reporting?); and (e) were any other sources of bias identified?

### Statistical analysis

Differences were expressed as relative risks (RRs) with 95% confidence intervals (CIs) for dichotomous outcomes and as weighted mean differences (WMDs) with 95% CIs for continuous outcomes. Heterogeneity across studies was tested by using the I^2 ^statistic, which was a quantitative measure of inconsistency across studies. Studies with an I^2 ^statistic of 25% to 50% were considered to have low heterogeneity, those with an I^2 ^statistic of 50% to 75% were considered to have moderate heterogeneity, and those with an I^2 ^statistic of greater than 75% were considered to have a high degree of heterogeneity [14]. An I^2 ^value of greater than 50% indicates significant heterogeneity [15]. A fixed-effects model was used, and a random-effects model was used in the case of significant heterogeneity (I^2 ^> 10%). Whenever heterogeneity was present, several sensitivity analyses were carried out to identify potential sources. We also investigated the influence of a single study on the overall pooled estimate by omitting one study in each turn. Owing to the limited number (below 10) of studies included in each analysis, publication bias was not assessed. A *P *value of less than 0.05 was considered statistically significant. Risk-of-bias assessment was conducted by using Review Manager version 5.0 (The Cochrane Collaboration, Software Update, Oxford, UK), and other statistical analyses were performed by using STATA version 11.0 (Stata Corporation LP, College Station, TX, USA).

## Results

### Study identification and selection

**Figure 1:**
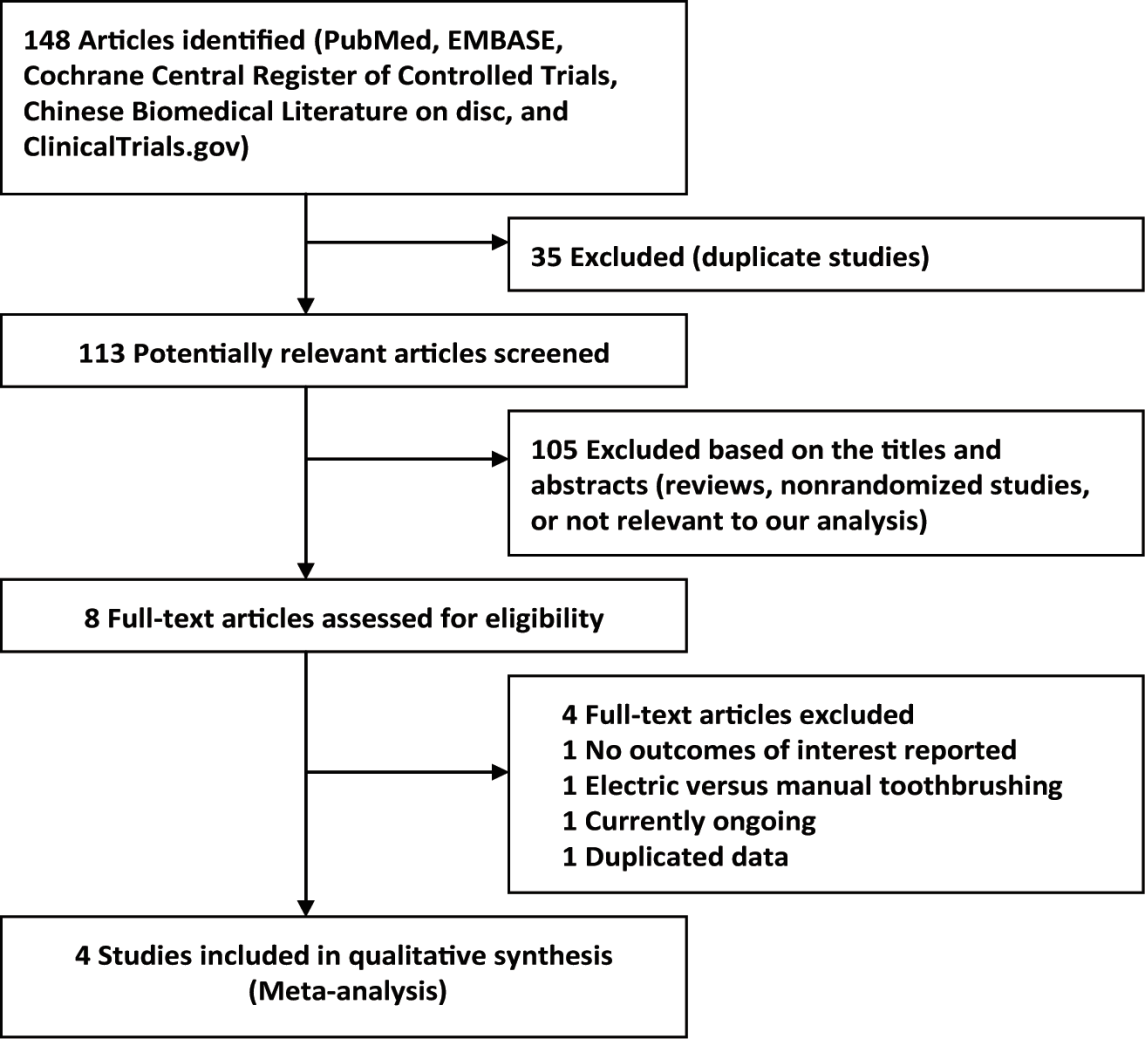
**Selection process for randomized controlled trials included in the meta-analysis**.

The initial search yielded 148 relevant publications, of which 140 were excluded for duplicate studies and various reasons (reviews, non-randomized studies, or not relevant to our analysis) on the basis of the titles and abstracts (Figure 1). The remaining eight were retrieved for full text review, and four of them were excluded because one did not report outcomes of interest [16], one pertained to electric rather than manual toothbrushing [17], one was currently ongoing [18], and one was duplicated data [19]. Thus, four RCTs were included in the final analysis [20-23].

### Study characteristics, quality, and bias assessment

**Figure 2:**
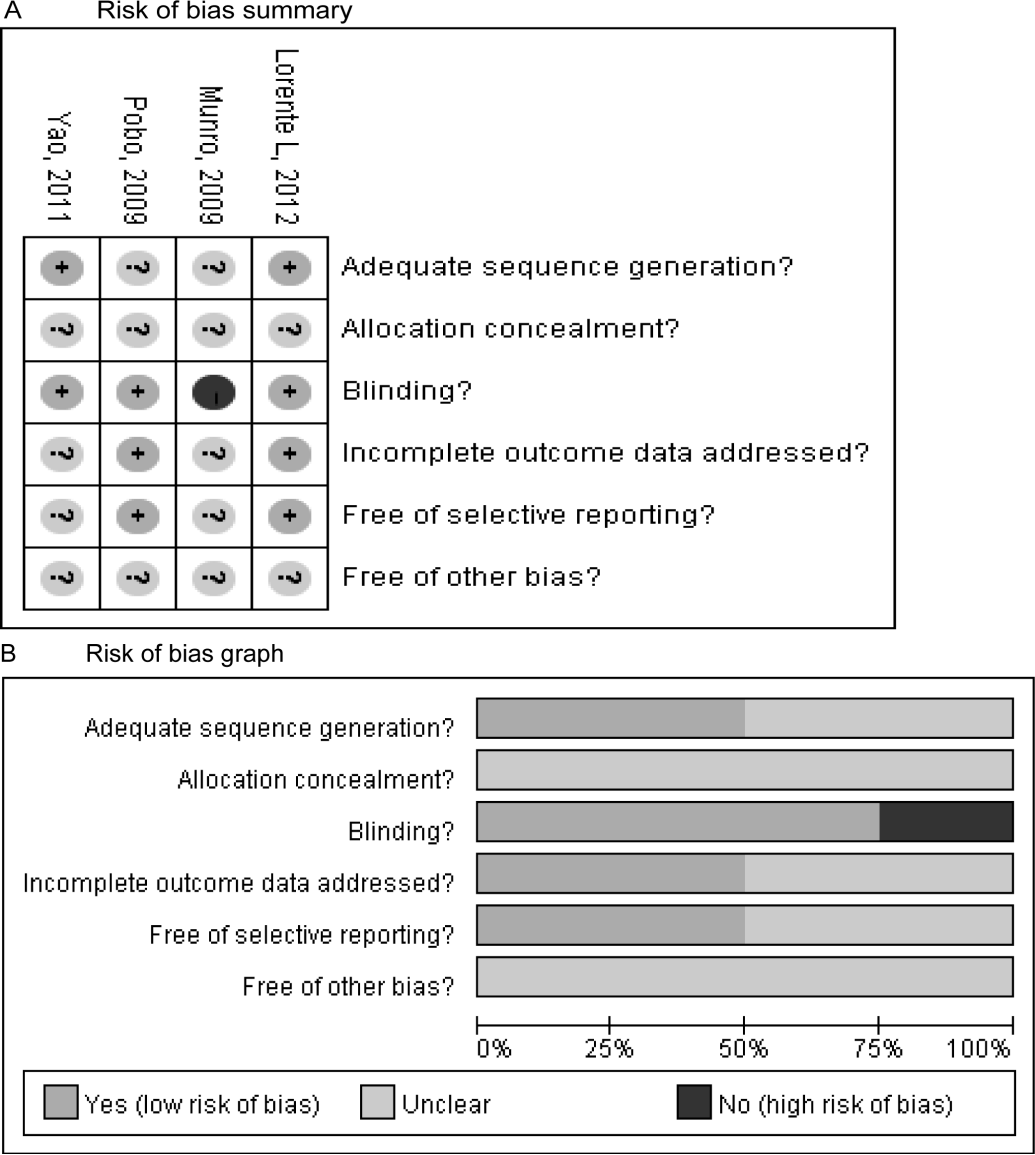
**Risk-of-bias analysis**. **(a) **Risk-of-bias summary: the authors' judgments about each risk-of-bias item for the included studies (Lorente *et al*. [23], Munro *et al*. [20], Pobo *et al*. [21], and Yao *et al*. [22]). **(b) **Risk-of-bias graph: the authors' judgments about each risk-of-bias item presented as percentages across all included studies.

The main characteristics of the four RCTs included in the meta-analysis are presented in Table 1 and the outcome data of each included trial are described in Table 2. These studies were published between 2009 and 2012. The sizes of the RCTs ranged from 53 to 436 patients (total of 828). The selected trials examined various populations in ICUs, including surgical [21], medical-surgical [20,22], and mixed (medical, surgical/trauma, and neuroscience) [23]. All of these patients received mechanical ventilation for more than 24 hours, and none had pneumonia. The definition of VAP varied across studies, and no standard definition was used in reported studies. The median Jadad score of the studies included was 3 (range of 2 to 3). Risk-of-bias analysis (Figure 2) revealed that only two of the included studies [22,23] adequately reported the randomization protocol and that none described a method used to conceal the allocation sequence in sufficient detail to exclude selection bias.

**Table 1:** Main characteristics of randomized controlled trials included in the meta-analysis of toothbrushing for ventilator-associated pneumonia prevention

Author/Year	Number of patients (I/C)	Type of ICU/Study population	Severity of illness (I/C)	Intervention group	Control group	Definition of VAP	Study design/Jadad score	Funding bias	Length of follow-up, days	Rate of successful follow-up
Munro *et al*. [20] (2009)	192 (97/95)	Medical-surgical/adult patients requiring MV > 24 hours, with no current pneumonia	APACHE III score: 76.4 ± 23.3/76.2 ± 3.3 and 76.2 ± 25.5/80.4 ± 28.7	0.12% CHX and toothbrushing (that is, soft pediatric toothbrush and toothpaste; brushing tooth by tooth, on anterior and posterior surfaces, the palate, and the tongue)	0.12% CHX 5 mL by oral swab twice daily or usual care	CPIS > 6	Non-blind, RCT/2	No	3	46%

Pobo *et al*. [21] (2009)	147 (74/73)	Surgical/adult patients requiring MV > 48 hours, with no current pneumonia	APACHE II score: 18.8 ± 7.1/18.7 ± 7.3	0.12% CHX and toothbrushing every 8 hours (that is, electric toothbrush; brushing tooth by tooth, on anterior and posterior surfaces, the gum line, and the tongue)	Oral care every 8 hours with 0.12% CHX	New or progressive pulmonary opacities together with purulent respiratory secretions plus fever > 38°C or leukocytosis > 10,000 cells/mL	Single-blind, RCT/3	No	28	100%

Yao *et al*. [22] (2011)	53 (28/25)	Medical-surgical/adult patients requiring MV > 48 to 72 hours, with no current pneumonia	APACHE II score: 19.6 ± 5.2/19.4 ± 4.4	Usual care using cotton swabs, elevating the head of the bed, moisturizing the lips, and before-and-after hypopharyngeal suctioning; toothbrushing (that is, electric and soft pediatric toothbrush; brushing tooth with purified water, teeth facial sides cleansed with electric toothbrush, and lingual sides, gums, mucosa, and tongue cleansed with pediatric toothbrush)	Usual care using cotton swabs, elevating the head of the bed, moisturizing the lips, and before-and-after hypopharyngeal suctioning	CPIS > 6	Single-blind, pilot, RCT/3	No	9	68%

Lorente *et al*. [23] (2012)	436 (217/219)	Medical, surgical/trauma, and neuroscience/adult patients requiring MV > 24 hours, with no current pneumonia	APACHE II score: 17.88 ± 8.84/19.16 ± 9.88	0.12% CHX and toothbrushing (that is, manually brushing tooth by tooth, on the anterior and posterior surfaces, the gum line, and the tongue for a period of 90 seconds)	Oral cleansing every 8 hours with 0.12% CHX	New onset of bronchial purulent sputum; body temperature > 38°C or < 35.5°C; white blood cell count > 10,000/mm^3 ^or < 4,000/mm^3^; chest radiograph showing new or progressive infiltrates; significant quantitative culture of respiratory secretions by tracheal aspirate (> 10^6 ^CFU/mL)	Single-blind, RCT/3	No	Not reported	100%

APACHE, Acute Physiology and Chronic Health Evaluation; CFU, colony-forming units; CHX, chlorhexidine; CPIS, clinical pulmonary infection score; I/C, intervention/control; ICU, intensive care unit; MV, mechanical ventilation; RCT, randomized controlled trial; VAP, ventilator-associated pneumonia.

**Table 2:** Outcome data of studies included in the meta-analysis of toothbrushing for ventilator-associated pneumonia prevention (intervention versus control)

Study	Primary outcome	Secondary outcomes				
	
	Incidence of VAP	ICU mortality	Length of ICU stay, days	Duration of MV, days	Antibiotic-free day, days	MV-free day, days
Munro *et al*. [20] (2009)	48/97 vs. 45/95	22/97 vs. 22/95	NR	NR	NR	NR

Pobo *et al*. [21] (2009)	15/74 vs. 18/73	16/74 vs. 23/73	12.9 ± 8.7 vs. 15.5 ± 9.6	8.9 ± 5.8 vs. 9.8 ± 6.1	7.6 ± 8.4 vs. 7.8 ± 7.6	9.5 ± 12.2 vs. 11.3 ± 12.3

Yao *et al*. [22] (2011)	4/28 vs. 14/25	NR	12.5 ± 6.1 vs. 13.5 ± 6.8	12.0 ± 11.0 vs. 13.6 ± 15.6	NR	NR

Lorente *et al*. [23] (2012)	21/217 vs. 24/219	62/217 vs. 69/219	12.07 ± 15.55 vs. 13.04 ± 17.27	9.18 ± 14.13 vs. 9.93 ± 15.39	7.43 ± 14.84 vs. 8.39 ± 16.83	4.03 ± 3.22 vs. 4.42 ± 3.93

ICU, intensive care unit; MV, mechanical ventilation; NR, not reported; VAP, ventilator-associated pneumonia.

### Primary outcome: ventilator-associated pneumonia

**Figure 3:**
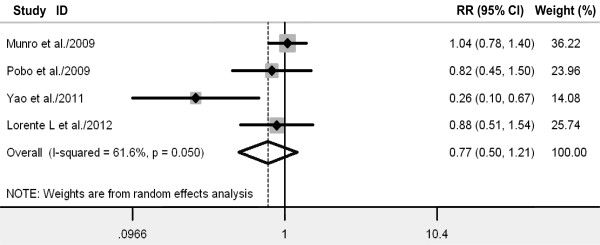
**Forest plot showing the effect of toothbrushing on the incidence of ventilator-associated pneumonia**. References cited are Munro *et al*. [20], Pobo *et al*. [21], Yao *et al*. [22], and Lorente *et al*. [23]. CI, confidence interval; RR, relative risk.

All four RCTs reported VAP in study patients. The aggregated results of these four studies suggest that oral care with toothbrushing was not associated with a significant reduction in the incidence of VAP (RR 0.77, 95% CI 0.50 to 1.21; *P *= 0.26) (Figure 3). The test for heterogeneity was significant (*P *for heterogeneity = 0.05; I^2 ^= 61.6%). Subsequently, we performed sensitivity analyses to explore potential sources of heterogeneity. Exclusion of the trial conducted by Yao and colleagues [22] resolved the heterogeneity but did not change the results (RR 0.95, 95% CI 0.75 to 1.22; *P *= 0.71; *P *for heterogeneity = 0.71; I^2 ^= 0%) [22]. Further exclusion of any single study did not materially alter the overall combined RR, which ranged from 0.64 (95% CI 0.34 to 1.20; *P *= 0.16) to 0.72 (95% CI 0.38 to 1.35; *P *= 0.31).

### Secondary outcomes

**Figure 4:**
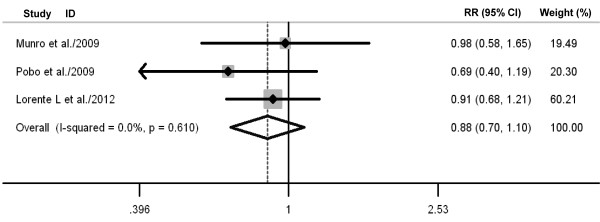
**Forest plot showing the effect of toothbrushing on intensive care unit mortality**. References cited are Munro *et al*. [20], Pobo *et al*. [21], and Lorente *et al*. [23]. CI, confidence interval; RR, relative risk.

**Figure 5:**
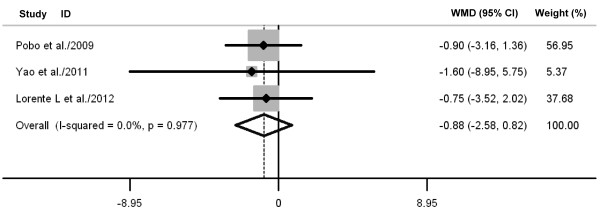
**Forest plot showing the effect of toothbrushing on duration of mechanical ventilation**. References cited are Pobo *et al*. [21], Yao *et al*. [22], and Lorente *et al*. [23]. CI, confidence interval; WMD, weighted mean difference.

**Figure 6:**
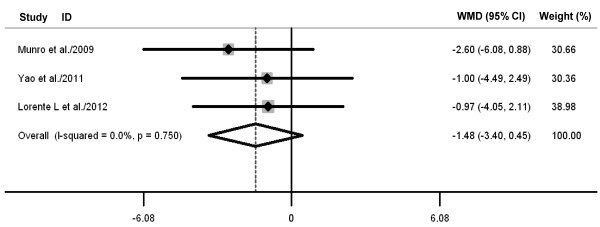
**Forest plot showing the effect of toothbrushing on length of intensive care unit stay**. References cited are Munro *et al*. [20], Yao *et al*. [22], and Lorente *et al*. [23]. CI, confidence interval; WMD, weighted mean difference.

**Figure 7:**
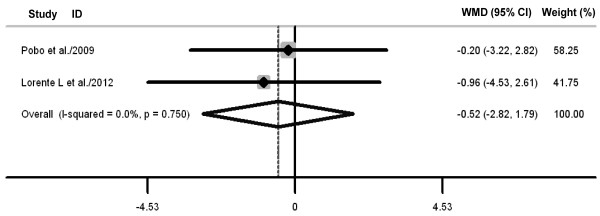
**Forest plot showing the effect of toothbrushing on antibiotic-free day**. References cited are Pobo *et al*. [21] and Lorente *et al*. [23]. CI, confidence interval; WMD, weighted mean difference.

**Figure 8:**
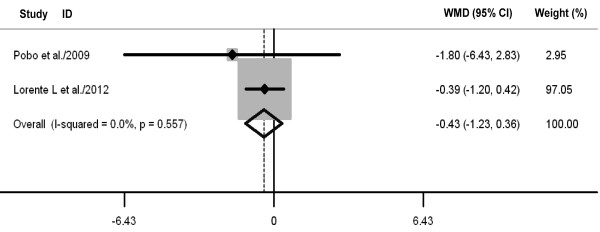
**Forest plot showing the effect of toothbrushing on mechanical ventilation-free day**. References cited are Pobo *et al*. [21] and Lorente *et al*. [23]. CI, confidence interval; WMD, weighted mean difference.

Oral care with toothbrushing was not associated with decreases in ICU mortality (three RCTs; RR 0.88, 98% CI 0.70 to 1.10; *P *= 0.26; *P *for heterogeneity = 0.61) (Figure 4), duration of mechanical ventilation (three RCTs; WMD -0.88 days, 95% CI -2.58 to 0.82; *P *= 0.31; *P *for heterogeneity = 0.98) (Figure 5), length of ICU stay (three RCTs; WMD -1.48 days, 95% CI -3.40 to 0.45; *P *= 0.13; *P *for heterogeneity = 0.75) (Figure 6), antibiotic-free day (two RCTs; WMD -0.52 days, 95% CI -2.82 to 1.79; *P *= 0.66; *P *for heterogeneity = 0.75) (Figure 7), and mechanical ventilation-free day (two RCTs; WMD -0.43 days, 95% CI -1.23 to 0.36; *P *= 0.29; *P *for heterogeneity = 0.56) (Figure 8). There was no evidence of heterogeneity for these secondary outcomes (all *P *values > 0.56; I^2 ^= 0%).

## Discussion

Our meta-analysis suggests that oral care with toothbrushing did not significantly reduce the incidence of VAP in adult critically ill patients receiving mechanical ventilation. In addition, oral care with toothbrushing was not associated with a markedly reduced ICU mortality, duration of mechanical ventilation, length of ICU stay, antibiotic-free day, or mechanical ventilation-free day.

Several high-quality non-randomized studies focusing on toothbrushing for VAP prevention are summarized in Table 3. All of them reported that oral care with toothbrushing was associated with a different degree of reduction in the incidence of VAP compared with no oral care [24-26]. However, the limitations of these studies are that non-randomized study design was used (case control [24] or pre/post-intervention observational study [25,26]). Moreover, it is not possible to discriminate the influence of toothbrushing alone, since oral care in the intervention group involved the simultaneous use of both other preventive measures (for example, an antibacterial agent) and toothbrushing.

**Table 3:** Summary of high-quality non-randomized studies on toothbrushing for ventilator-associated pneumonia prevention

Study	Type of trial	Patient characteristics	Methods	Results
Mori *et al*. [24] (2006)	Non-randomized trial with historical controls (case control)	Medical-surgical ICU; 1,666 adult patients requiring MV ≥48 hours	Study compared two groups: (a) historical controls (n = 414) who received no systematic oral care and (b) intervention group (n = 1,252) that received oral care three times a day. A written protocol directed oral care that included toothbrushing and rinses with povidone-iodine three times daily.	Incidence of VAP (per 1,000 ventilator days) in the oral care group was significantly lower than that in the non-oral care group (3.9 versus 10.4). Results showed decreased incidence of VAP in the oral care group.

Garcia *et al*. [25] (2009)	Pre/post-intervention observational study	Medical ICU; 1,538 adult patients requiring MV ≥48 hours	Study compared two groups: (a) controls (n = 779): before the intervention had no oral procedures (for example, oral assessments, suctioning of subglottic space, or toothbrushing) and (b) intervention (n = 759): during the intervention period had oral care techniques. Oral care consisted of oral assessment, deep suctioning every 6 hours, oral cleaning every 4 hours, and toothbrushing twice daily.	Incidence of VAP (per 1,000 ventilator days) in the oral care group was significantly lower than that in the non-oral care group (8 versus 12). Results showed decreased incidence of VAP in the oral care group. Mortality and length of ICU stay were also reduced significantly.

Sona *et al*. [26] (2009)	Pre/post-intervention observational study	Surgical ICU; 1,648 adult patients requiring MV	Study compared (a) controls (n = 777): during the preintervention period and (b) intervention (n = 871): after institution of oral care interventions. Oral care protocol included toothbrushing for 1 or 2 minutes at 12-hour intervals with sodium monofluorophosphate 0.7% paste. Used stock toothbrush. Applied 15 mL of 0.12% chlorhexidine solution.	Incidence of VAP (per 1,000 ventilator days) in the oral care group was significantly lower than that in the non-oral care group (2.4 versus 5.2). Results showed decreased incidence of VAP in the oral care group.

ICU, intensive care unit; MV, mechanical ventilation; VAP, ventilator-associated pneumonia.

The principal finding of our meta-analysis seems to contradict the aforementioned studies on the topic. In particular, the present meta-analysis included four RCTs involving 828 patients and indicated that oral care with toothbrushing was not associated with a reduction in the incidence of VAP in critically ill patients receiving mechanical ventilation. Substantial heterogeneity was observed among these studies, and this was not surprising given the differences in characteristics of populations, oral care protocols, and study designs. Our sensitivity analyses suggest that the trial conducted by Yao and colleagues [22] probably contributed to the heterogeneity. This study differed from the others in some aspects. On one hand, this trial adopted oral care protocols without chlorhexidine; on the other hand, the small number of cases and participants increased the possibility that chance accounted for the results.

Our study provides additional interesting clues that may be useful for future research on the topic. Remarkably, the study conducted by Yao and colleagues [22] included in our meta-analysis used unique oral care protocols. Unlike other trials, that study did not include chlorhexidine and found that an oral care protocol of toothbrushing with purified water can effectively reduce the incidence of VAP and improve oral health and hygiene. Thus, one may focus on this specific oral care protocol (toothbrushing alone without chlorhexidine) to better address the isolated effect of toothbrushing. More large-scale and well-performed RCTs are warranted.

Our meta-analysis showed that oral care with toothbrushing did not alter other important clinical outcomes, including ICU mortality, duration of mechanical ventilation, length of ICU stay, antibiotic-free day, and mechanical ventilation-free day. These results are not conclusive inasmuch as further adequately powered studies are needed. In fact, these included studies are not adequately powered to examine these secondary outcome measures since they were not the primary outcomes and were the only clinically significant endpoints consistently reported in many of the studies analyzed in the present meta-analysis. Further studies should pay more attention to these clinical endpoints other than just the incidence of VAP.

Most of the included RCTs did not report complications of toothbrushing during the study period. Toothbrushing may give rise to a number of complications, such as the appearance of oral bleeding in patients with severe coagulopathy because of the application of greater force than when applied by the patient. In addition, the action of toothbrushing could facilitate the accidental removal of the endotracheal tube, with the need for reintubation, and this fact has been associated with VAP in some studies [27-29]. Another concern was the risk of bacteremia after toothbrushing [30].

Some limitations of this meta-analysis should be taken into account. First, our analysis is based on only four RCTs and some of them have a modest sample size. Overestimation of the treatment effect is more likely in smaller trials compared with larger samples. Second, there was considerable heterogeneity among the included trials. The targeted population varied greatly. The adopted oral care protocols, definition of VAP, and study designs differed. These factors may result in the heterogeneity and have a potential impact on our results. Furthermore, because of the limited number of RCTs regarding the secondary outcomes, caution should be taken when interpreting the results. Finally, it was possible that the exclusion of some missing and unpublished data led to bias in effect size.

## Conclusions

Despite its various limitations, our study still is clinically valuable because it suggests that oral care with versus without toothbrushing does not significantly reduce the incidence of VAP and alter other important clinical outcomes in mechanically ventilated patients. On the basis of these findings, there is currently a lack of evidence to support toothbrushing in patients receiving mechanical ventilation. However, relevant evidence is still limited but is accumulating. Thus, further large-scale, well-designed RCTs are urgently needed.

## Key messages

• Oral care with toothbrushing does not significantly reduce the incidence of ventilator-associated pneumonia and alter other important clinical outcomes in mechanically ventilated patients.

• There is currently a lack of evidence to support toothbrushing in patients receiving mechanical ventilation.

• Larger adequately powered randomized controlled trials are warranted to clarify the isolated effect of toothbrushing on the prevention of ventilator-associated pneumonia.

## Abbreviations

CI: confidence interval; ICU: intensive care unit; RCT: randomized controlled trial; RR: relative risk; VAP: ventilator-associated pneumonia; WMD: weighted mean difference.

## Competing interests

The authors declare that they have no competing interests.

## Authors' contributions

W-JG conceived the study, participated in the design, collected the data, and drafted the manuscript. LP collected the data and performed statistical analyses. Y-XN helped to collect the data. Y-ZG and J-CL conceived the study, participated in the design, and helped to draft the manuscript. All authors read and approved the final manuscript.
